# Ecological and socio-economic functions across tropical land use systems after rainforest conversion

**DOI:** 10.1098/rstb.2015.0275

**Published:** 2016-05-19

**Authors:** Jochen Drescher, Katja Rembold, Kara Allen, Philip Beckschäfer, Damayanti Buchori, Yann Clough, Heiko Faust, Anas M. Fauzi, Dodo Gunawan, Dietrich Hertel, Bambang Irawan, I. Nengah S. Jaya, Bernhard Klarner, Christoph Kleinn, Alexander Knohl, Martyna M. Kotowska, Valentyna Krashevska, Vijesh Krishna, Christoph Leuschner, Wolfram Lorenz, Ana Meijide, Dian Melati, Miki Nomura, César Pérez-Cruzado, Matin Qaim, Iskandar Z. Siregar, Stefanie Steinebach, Aiyen Tjoa, Teja Tscharntke, Barbara Wick, Kerstin Wiegand, Holger Kreft, Stefan Scheu

**Affiliations:** 1 Johann-Friedrich-Blumenbach Institute for Zoology and Anthropology, University of Göttingen, Berliner Strasse 28, 37073 Göttingen, Germany; 2 Biodiversity, Macroecology and Conservation Biogeography, University of Göttingen, Büsgenweg 1, 37077 Göttingen, Germany; 3 Bioclimatology, Büsgen Institute, University of Göttingen, Büsgenweg 2, 37077 Göttingen, Germany; 4 Soil Science of Tropical and Subtropical Ecosystems, Büsgen Institute, University of Göttingen, Büsgenweg 2, 37077 Göttingen, Germany; 5 Chair of Forest Inventory and Remote Sensing, University of Göttingen, Büsgenweg 5, 37077 Göttingen, Germany; 6 Department of Plant Protection, Bogor Agricultural University, Kampus IPB Darmaga, Bogor 16680, Indonesia; 7 Department of Agroindustrial Technology, Bogor Agricultural University, Kampus IPB Darmaga, Bogor 16680, Indonesia; 8 Forest Resources Inventory and Remote Sensing, Bogor Agricultural University, Kampus IPB Darmaga, Bogor 16680, Indonesia; 9 Department of Silviculture, Bogor Agricultural University, Kampus IPB Darmaga, Bogor 16680, Indonesia; 10 Agroecology, Department of Crop Sciences, University of Göttingen, Grisebachstrasse 6, 37077 Göttingen, Germany; 11 Centre for Environmental and Climate Research, Lund University, Sölvegatan 37, 22362 Lund, Sweden; 12 Department of Human Geography, University of Göttingen, Goldschmidtstrasse 5, 37077 Göttingen, Germany; 13 Centre for Climate Change and Air Quality, Agency for Meteorology, Climatology and Geophysics (BMKG), Jln Angkasa I No. 2, Jakarta 10720, Indonesia; 14 Department of Plant Ecology and Ecosystem Research, Albrecht-von-Haller Institute for Plant Sciences, University of Göttingen, Untere Karspüle 2, 37073 Göttingen, Germany; 15 Faculty of Forestry, University of Jambi, Jln Raya Jambi-Muara Bulian km 15, Mendalo Darat, Jambi 36361, Indonesia; 16 Department of Agricultural Economics and Rural Development, University of Göttingen, Platz der Göttinger Sieben 5, 37073 Göttingen, Germany; 17 Graduate School of Life Sciences, Tokohu University, Aroba 6-3, Aramaki, Aoba-ku, Sendai 980-85478, Japan; 18 Institute of Social and Cultural Anthropology, University of Göttingen, Theaterplatz 15, 37073 Göttingen, Germany; 19 Agriculture Faculty of Tadulako University, Jln Soekarno Hatta km 09, Tondo, Palu 94118, Indonesia; 20 Ecosystem Modelling, University of Göttingen, Büsgenweg 4, 37077 Göttingen, Germany

**Keywords:** agroforestry, biodiversity and ecosystem function, deforestation, EFForTS, oil palm, jungle rubber

## Abstract

Tropical lowland rainforests are increasingly threatened by the expansion of agriculture and the extraction of natural resources. In Jambi Province, Indonesia, the interdisciplinary EFForTS project focuses on the ecological and socio-economic dimensions of rainforest conversion to jungle rubber agroforests and monoculture plantations of rubber and oil palm. Our data confirm that rainforest transformation and land use intensification lead to substantial losses in biodiversity and related ecosystem functions, such as decreased above- and below-ground carbon stocks. Owing to rapid step-wise transformation from forests to agroforests to monoculture plantations and renewal of each plantation type every few decades, the converted land use systems are continuously dynamic, thus hampering the adaptation of animal and plant communities. On the other hand, agricultural rainforest transformation systems provide increased income and access to education, especially for migrant smallholders. Jungle rubber and rubber monocultures are associated with higher financial land productivity but lower financial labour productivity compared to oil palm, which influences crop choice: smallholders that are labour-scarce would prefer oil palm while land-scarce smallholders would prefer rubber. Collecting long-term data in an interdisciplinary context enables us to provide decision-makers and stakeholders with scientific insights to facilitate the reconciliation between economic interests and ecological sustainability in tropical agricultural landscapes.

## 1. Introduction

Growing human population and rising *per capita* consumption lead to ecosystem degradation and biodiversity decline worldwide [1–5]. Agricultural expansion for the production of food, feed, fibre and fuel has generated fundamental benefits for human welfare (e.g. [6]) but comes with a variety of costs [7,8]. These costs may compromise human well-being in the long term [9], as they are linked to greenhouse gas emissions [10], declining biodiversity [11,12] and degradation of a variety of regulatory ecosystem services that affect air quality, purification of water, carbon storage or soil erosion [13–15]. It is commonly assumed that the conversion of natural to agricultural systems leads to major losses of important ecosystem services [16]. However, the degree to which agricultural systems still provide certain ecosystem services strongly depends on the converted ecosystem, the type of planted crop, the spatial dimensions of plantations, and the management practices in place [17]. With the exception of small-scale experiments, the mechanisms governing the relationship between biodiversity and ecosystem functions (BEF, [3]) remain poorly understood [18], especially in tropical rainforest ecosystems, which experience massive transformations and varying land use intensities [19]. In order to understand BEF relationships in rainforest transformation systems as well as to facilitate the reconciliation of economic interests and ecological sustainability therein, detailed ecological and socio-economic evaluations of these systems are needed, both at different spatial and temporal scales as well as under different institutional conditions [20,21].

In tropical Asia, a rapidly growing population [22,23] and agricultural expansion coincides with one of the highest levels of biodiversity and endemism worldwide [24,25]. Rainforests in Southeast Asia have been logged on a large scale since the mid 20th century, usually followed by subsequent transformation of logged-over rainforests into cash crop monocultures [26,27], such as acacia, rubber and oil palm. In Indonesia, this process has accelerated during the past decades, where annual loss in rainforest cover was estimated at 0.84 million hectares for the year 2012, the highest worldwide [28]. Large-scale conversion of rainforest for agricultural use is particularly evident on the island of Sumatra, which experiences the highest primary rainforest cover loss in all of Indonesia [16,28,29].

**Figure 1.**
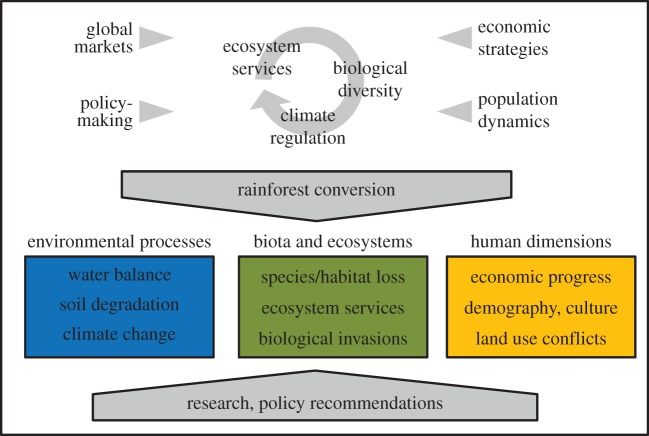
Conceptual approach of the EFForTS project. EFForTS combines research on environmental processes, biota and ecosystems, and human dimensions to understand the drivers and consequences of current agricultural development in Jambi Province (Indonesia).

In the Ecological and Socio-economic Functions of Tropical Lowland Rainforest Transformation Systems project (EFForTS project, http://www.uni-goettingen.de/crc990), we comprehensively study the environmental processes as well as the ecological and socio-economic dimensions of the current agricultural transformation processes in Jambi Province (figure 1). Four research foci serve as a basis for the synthesis of this interdisciplinary project:

(1) Assessing the ecological and socio-economic functions across rainforest transformation systems;
(2) Quantifying the effects of spatial and temporal variability on ecological and socio-economic functions;
(3) Scaling-up of ecological and socio-economic functions from local to landscape and regional scales and
(4) Contributing to approaches towards more sustainable land use in tropical regions.

This long-term research project aims at an in-depth understanding of the drivers and consequences of rainforest transformation into agricultural landscapes for biodiversity, ecosystem functions and human well-being.

## 2. Study area

**Figure 2.**
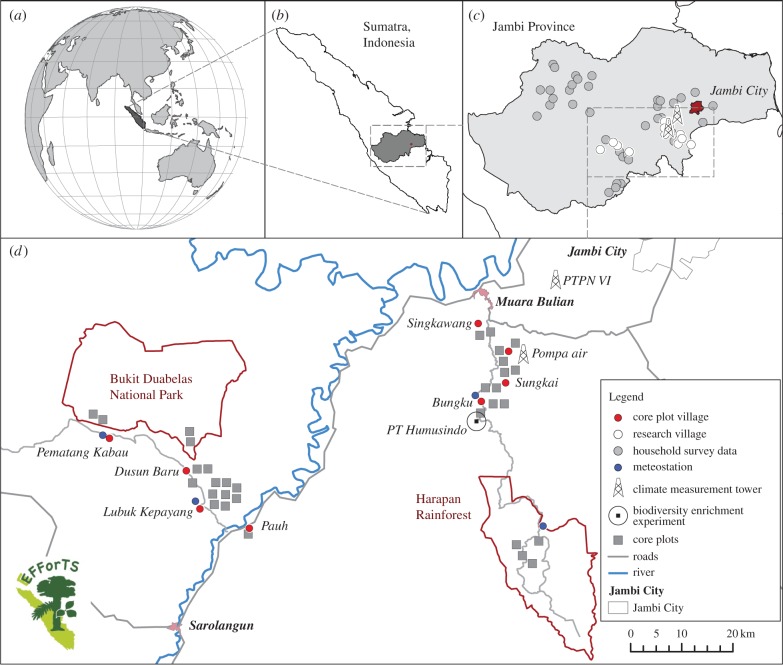
Location of EFForTS study sites in Sumatra (*a,b*) and Jambi Province (*c,d*). Socio-economic surveys are carried out all over Jambi Province (*c*), while the core plot design is located in two landscapes near to Bukit Duabelas National Park and Harapan Rainforest (*d*).

**Figure 3.**
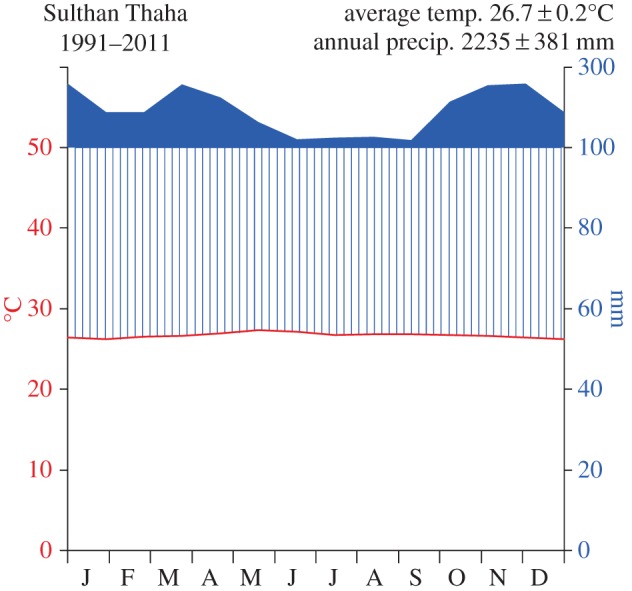
Average monthly temperature and rainfall at Sulthan Thaha Airport, Jambi City, from 1991 to 2011 (Sulthan Thaha Airport station). The relation of average monthly rainfall (solid and striped blue) to average monthly temperature (red line) illustrates Jambi's humid climate, with mean monthly rainfall above 100 mm throughout the year.

**Figure 4.**
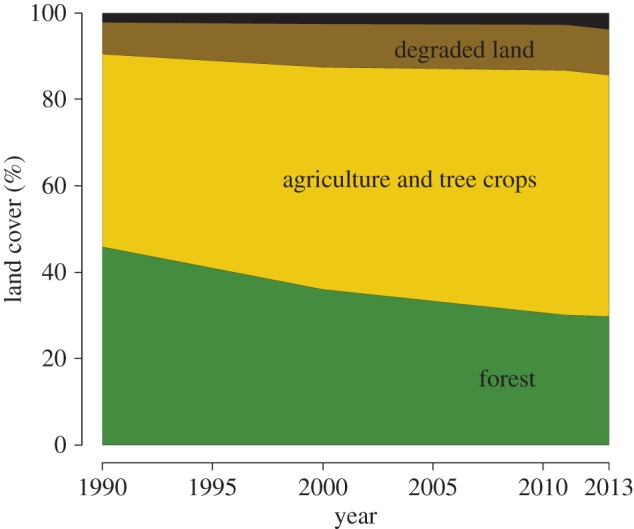
Land cover in Jambi Province from 1990 to 2013. Data were obtained by a series of Landsat imageries with spatial resolution of 30 × 30 m. The entire area of Jambi Province was used as reference. Degraded land (brown): shrubs, bare land, burnt areas; others (black): settlements, water bodies, fishpond.

EFForTS conducts research in Jambi Province in Sumatra (Indonesia, figure 2). Jambi Province covers a land area of 50 160 km^2^ [30] and stretches from the Barisan mountain range in the west across extensive lowlands towards the southern Malacca Strait in the east. The climate in Jambi's lowlands is tropical humid with two peak rainy seasons around March and December, and a dryer period during July–August (figure 3). Jambi's rainforests have a long history of exploitation, including traditional agroforestry systems and the extraction of timber and non-timber products [31,32]. The first large commercial logging concessions were issued in the 1970s [33]. Since then, governmental land and resource use policies have been combined with population migration schemes to foster economic development [34,35]: rainforests, often previously logged, have been converted into intensively managed agro-industrial production zones to grow cash crop trees of rubber (*Hevea brasiliensis*) and oil palm (*Elaeis guineensis*) or fast-growing tree species such as *Acacia mangium* for pulp production. From 1967 to 2007, roughly 400 000 people were resettled in a governmental transmigration programme from densely populated regions like Java to Jambi Province [36], who then mainly engaged in cash crop production. As of 2014, more than 650 000 ha of rubber and more than 590 000 ha of oil palm are being cultivated in Jambi Province [30]. Increasing population and agricultural activity led to rapid land-cover changes in Jambi resulting in a continuous decrease of rainforest cover. In 2013, only 30% of Jambi Province was covered with rainforest (mainly located in mountainous areas), while 55% was already converted into agricultural land, and 10% of the land was degraded/fallow (mainly comprising land awaiting conversion into monocultures; figure 4).

## 3. Study design

Rainforest conversion entails a variety of abiotic, biotic and socio-economic changes [20]. EFForTS is thus based on three major lines of research: (i) environmental processes, (ii) biota and ecosystem services, and (iii) human dimensions (figure 1). The data for environmental processes and biota and ecosystem services presented here were collected in the ‘core plot design’, while data on human dimensions were collected in the ‘socio-economic survey design’.

For the core plot design, we established research core plots in four different land use systems (lowland rainforest, jungle rubber, rubber monoculture and oil palm monoculture) in two landscapes, the ‘Bukit Duabelas landscape’ and the ‘Harapan landscape’ (figure 2). Rainforest core plots represent ‘primary degraded forest’ as classified by Margono *et al.* [28] and show signs of selective logging and extraction of non-timber rainforest products. Jungle rubber represents a smallholder rubber agroforest system which is established by planting rubber trees into (often previously logged) rainforests [37]. All rubber and oil palm core plots have been established in smallholder monoculture plantations, which varied in age between 7 and 16 years for rubber and between 8 and 15 years for oil palm at the time of plot selection in 2012. All core plots were established on Acrisol soils. While soils in the Harapan landscape contain more even fractions of sand, silt and clay (loam Acrisols), soils in the Bukit Duabelas landscape are clay Acrisols, characterized by higher proportions of clay [38]. In each landscape, we established four core plots in each of the four land use systems, resulting in a total of 32 plots (electronic supplementary material, table S1). Each core plot measures 50 × 50 m and contains five 5 × 5 m subplots at fixed positions that were randomly assigned (electronic supplementary material, figure S1). Each core plot is equipped with a meteorological station which measures air temperature, relative air humidity, soil temperature and soil moisture. The core plot design is extended by a biodiversity enrichment experiment and a meteorological monitoring network (see the electronic supplementary material, text S2).

The ‘socio-economic survey design’ follows a complementary approach ranging from household (micro) and village (meso) to national and international (macro) level using a joint sampling framework (for details see [39], electronic supplementary material, figure S2). This study mainly uses data from village and household surveys, conducted during August–December 2012. The socio-economic village survey covers 93 randomly selected and five purposively selected villages. The latter come from the two core plot landscapes to allow linking of ecological, environmental and socio-economic data and integrated modelling. The household survey includes 700 farmer households, of which 600 reside in the randomly selected villages and 100 in the purposively selected ones.

Within the core plot and socio-economic design, a variety of data were collected covering soil, water, atmosphere, biogeochemical cycles, biomass, carbon stocks, above- and below-ground biodiversities, community structure, food web dynamics, energy and nutrient fluxes as well as economic (e.g. profit, income, employment), social (e.g. income distribution, risk, poverty, food security, culture, gender) and institutional (e.g. sharecropping, property rights) factors. In the following, we present key results and respective methods used to analyse ecosystem functions, biodiversity of plants, above- and below-ground invertebrates and key socio-economic characteristics.

## 4. Material and methods

### (a) Temperature and humidity

Below-canopy air temperature and relative humidity were measured hourly by meteorological stations within each core plot and stored using a UIT LogTrans 16-GPRS data logger. The meteorological stations were equipped with thermohygrometers (Galltec Mela^®^) placed at a height of 2 m to record air temperature (°C) and humidity (%), and additional soil sensors (IMKO TRIME-PICO) at 0.3 m depth to monitor soil temperature (°C) and moisture (vol %).

### (b) Canopy openness

Canopy openness was derived from hemispherical photographs taken at 1.2 m above the ground from 32 positions within each core plot (Canon EOS 700D SLR camera and SIGMA 4.5 mm F2.8 EX DC circular fisheye lens). A bubble level slotted into the flash socket, vertically levelled the camera and aligned it to the magnetic north. Exposure was determined by following a histogram-exposure protocol [40] and photographs were processed by applying the ‘Minimum’ thresholding algorithm’ [41] using ‘ImageJ’ [42].

### (c) Leaf litterfall

Leaf litterfall was measured monthly from March 2013 to April 2014 by placing 16 litter traps (75 × 75 cm) in each core plot. Collected leaves were sorted and oven dried at 60°C for 72 h. Oil palm fronds are pruned with each harvest as a standard management procedure. We measured the dry weight of 16 fully grown palm fronds (two per core plot in oil palm plantation) and counted all pruned palm fronds during harvest, thus allowing to calculate average ‘litterfall’ in oil palm plantations per area and time.

### (d) Litter carbon

To determine the amount of litter in the litter layer, five cores of a diameter of 5 cm were taken from each plot and pooled, resulting in 32 samples. The material was dried at 65°C for 72 h and weighed. Aliquots of the material were milled and analysed for total C concentrations using an elemental analyser (Carlo Erba, Milan, Italy). From these data, the total amount of C per area was calculated.

### (e) Tree biomass

Height and diameter at breast height (DBH) of all trees and palms in the core plots were recorded. Wood density was determined for cores of 208 trees (10 each in rainforest and jungle rubber plots, five each in rubber plantations). Interpolated values were applied for the remaining trees based on a calibration equation with pin penetration depth. Rainforest understorey trees with a diameter of 2–10 cm were inventoried in the same way on two subplots in each plot. Above-ground biomass, coarse-root and root stock biomass were modelled using standard allometric equations [43–47]. Fine-root biomass was assessed separately using 10 vertical soil cores (3.5 cm in diameter) down to 50 cm soil depth including the organic layer on each plot, from which all fine-root segments longer than 1 cm were extracted. The C concentration of all components (stem wood, fine roots and leaf litter) was analysed with a CN Analyser (Vario EL III, Hanau, Germany).

### (f) Plant species richness

Plant species numbers include all trees with a DBH ≥ 10 cm within the entire core plot, plus all vascular plant species found within the five subplots nested within each core plot.

### (g) Ant species richness

Arboreal arthropods were sampled from three locations in each of the 32 core plots by canopy fogging. We used DECIS 25 (Bayer CropScience) mixed with petroleum-based white oil in a 9 : 1 ratio (white oil : DECIS25) forming a visible fog which allows directing the fog towards the target canopies. Target canopies consisted of mixed tree canopies in rainforest and jungle rubber, two trees in rubber plantations, and one palm in oil palm plantations. Paralysed and dead arthropods were collected in 16 funnels of 1 m² per replicate; each funnel was fitted with a plastic bottle containing 96% EtOH. Immediately after sampling, the specimens were cleaned of debris, washed, EtOH exchanged, and stored at −20°C.

### (h) Oribatid mite species richness

Oribatid mites (Oribatida, Acari) were extracted from soil cores of 16 × 16 cm taken from each core plot with a spade. Litter and top soil layers (to a depth of 5 cm) of each sample were extracted separately using the high-gradient canister method in modified Kempson extractors [48].

### (i) Labour and gross margin

Labour intensity (’00 h ha^−1^ yr^−1^), gross margin per land unit (million IDR ha^−1^ yr^−1^) and gross margin per work hour (‘0000 IDR h^−1^) are based on the socio-economic survey of 700 farmer households [39]. These data are available for all three transformation systems except rainforest, i.e. jungle rubber, rubber plantations and oil palm plantations.

## 5. Results and discussion

**Figure 5.**
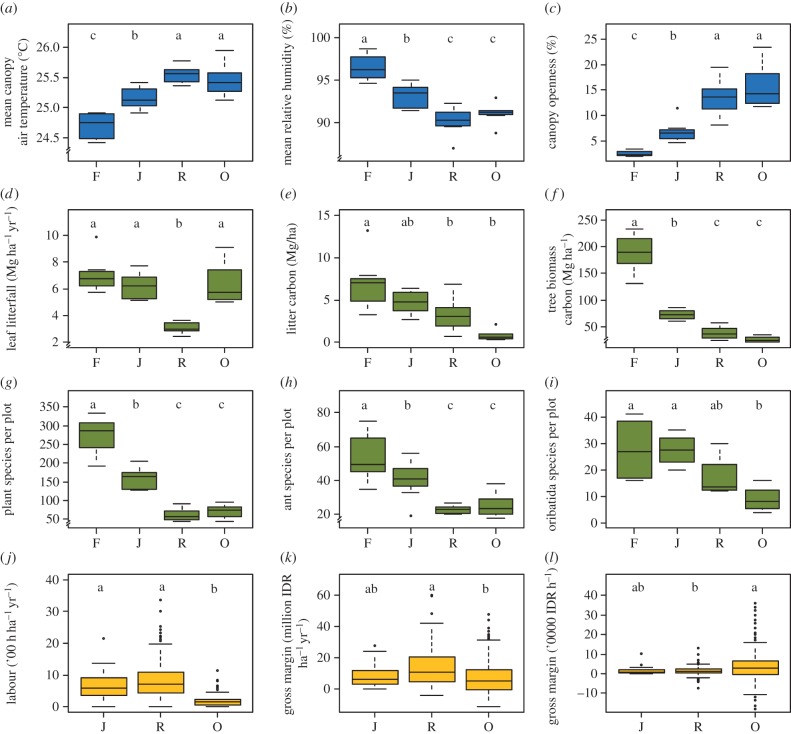
Environmental (blue), ecological (green) and socio-economic (yellow) differences among rainforest (F), jungle rubber (J), rubber (R), and oil palm (O) plantations. Three outliners are not shown: (k) 7100 rubber labour hours, (l) –52.2 and –25.6 oil palm gross margin/labour. Letters indicate significant differences between land use systems ((*a–i*) ANOVA, Tukey's HSD test, *p* < 0.05; (*j–l*) Kruskal–Wallis/Kruskalmc test, *p* < 0.05) (for details, see the electronic supplementary material).

Data from the core plots and socio-economic surveys after 3 years of measurement reveal marked differences in key factors and processes between the four land use systems (figure 5). Stand microclimate, vegetation structure, biodiversity and carbon fluxes differ significantly between rainforest and monocultures of rubber and oil palm, while jungle rubber often takes an intermediate position. Rainforest was characterized by lower mean air temperature and higher humidity compared with the other land use systems (figure 5*a,b*), corresponding to a denser canopy (figure 5*c*). Litterfall was significantly lower in rubber monoculture than in the other land use systems (figure 5*d*) and carbon stored in the litter layer was highest in rainforest (figure 5*e*). It is important to note that litterfall in oil palm plantations does not occur naturally, but leaves are cut during fruit harvest and piled up in rows between oil palms. Heterogeneous litter distribution in oil palm may lead to the apparent discrepancy between high leaf litter input (figure 5*d*) but low litter carbon stock (figure 5*e*) in oil palm, as the latter was not measured in piles of palm fronds. The decrease of leaf litter from rainforest to jungle rubber, rubber plantation and oil palm plantations may explain the significant decrease in species richness, density and biomass of leaf litter invertebrates, reducing energy fluxes from rainforest to oil palm communities by up to 51% [49]. Rainforests contained more than twice as much above- and below-ground tree biomass carbon as jungle rubber, and more than four times as much as the monoculture plantations (figure 5*f*, see also [50]).

Species richness in the agricultural land use systems was significantly lower than in rainforests. This is particularly apparent in vascular plants, where rainforest had almost six times as many species as the monocultures (figure 5*g*). Previous research suggests that plant diversity is a reliable predictor of arthropod diversity in the tropics [51]. This is confirmed by our data, where above-ground consumer species richness (here: canopy ants) was about twice as high in rainforest as in the converted ecosystems (figure 5*h*). For both vascular plants and canopy ants, there is a clear shift from communities dominated by native species in rainforest and jungle rubber towards communities dominated by introduced species in rubber and oil palm plantations (J. Drescher & K. Rembold, personal observation 2015). Species richness of below-ground consumers (here: oribatid mites) shows a similar but less pronounced decline from rainforest towards the other land use systems.

The three agricultural land use systems, jungle rubber, rubber plantation and oil palm plantation, differ markedly in key socio-economic properties. Comparing the plantation crops, oil palm required significantly less labour per hectare than jungle rubber and rubber monocultures (figure 5*j*), but generated relatively lower gross margin per unit area (figure 5*k*). However, the gross margin per unit of labour was highest in oil palm (figure 5*l*). Hence, when labour is limited, farmers have an incentive to grow oil palm; farmers for whom land is the scarcest factor in turn have an incentive to grow rubber. Interestingly, jungle rubber has a similar gross margin as rubber monoculture per unit of labour (lower than oil palm), but is not competitive with rubber monocultures per unit of land, which drives the intensification of jungle rubber into rubber plantations. In 2012, about 50% of the land in the surveyed villages was rubber, including both jungle rubber and monoculture plantations, while 12% was cultivated with oil palm (an increase from 5.0% 10 years earlier, with an increase of 8.5% for the entire study area during the same period). Most of the remaining village land was covered by rainforest (17%) at various stages of degradation or by fallow land (15%). Fallow land often represents degraded land dominated by shrubs after clear-cutting in preparation for agricultural use. While most of the plantations and fallow lands in the villages are held and managed by individual smallholder households, the rainforest land within village boundaries is usually managed by the state and only occasionally by the community. Smallholders in Jambi Province grow hardly any food crops, except for certain villages near urban centres. The socio-economic surveys revealed that the recent and ongoing transformation of land use systems other than oil palm started among the migrant population in the late 1980s, whereas the local population has engaged in oil palm cultivation only since the mid 1990s. Case study interviews revealed that land use change in Jambi is largely founded on different layers of past and present land rights which provoke the present controversy of land use, resource exploitation and the socio-economic consequences of these. The core drivers of land use change in Jambi are private and public investment, federal development schemes (i.e. for migrants) and the national Indonesian policy of resource exploitation, all of which are fuelled by the international demand for agrarian commodities such as rubber and palm oil.

Together, these first results from this extensive research project demonstrate that the conversion of rainforest into rubber and oil palm monocultures leads to substantial losses in plant and animal diversities, reduces above- and below-ground carbon stocks to a fraction of their original state, and significantly alters microclimatic conditions (figure 5). In-depth studies further showed that this conversion process also reduces energy fluxes, decreases soil fertility and increases soil erosion [38,49,52]. While rainforest conversion into monocultures has clearly negative effects on the environment, biota and ecosystems, many smallholders benefit substantially from the higher economic value of oil palm and rubber plantations [53,54]. Therefore, it is not surprising that rural population growth has a strong impact on the ongoing deforestation [55]. The economic benefits of this transformation process, however, are mainly experienced by landowners, while people without land experience various disadvantages from land use intensification towards monoculture cash crops, e.g. due to increasing food prices [26].

This study delivered vital baseline data for the long-term monitoring of BEF dynamics after rainforest conversion into rubber and oil palm plantations. In order to be able to provide evidence-based policy recommendations, however, complementary studies are necessary which go beyond recording the status quo. Specifically, experimental studies targeting land-sparing and land-sharing approaches [56,57] might provide realistic solutions which could then be presented to policy-makers. While the running biodiversity enrichment experiment (electronic supplementary material, Text S2) tests a land-sharing approach in oil palm, future EFForTS data collection will—among others—focus on rainforest conversion and rubber/oil palm agriculture on waterlogged soils of low productivity [58,59], thus analysing potentials for land sparing in arable landscapes. Together, running long-term data collections and experimental approaches will provide us with a detailed understanding of the interactions and feedback loops between humans, nature and environment in growing oil palm- and rubber-dominated landscapes, and hold the key to the reconciliation of conservation needs and socio-economic development in Southeast Asia.

## Supplementary Material

Electronic Supplemental Material (ESM)

## Data accessibility

The datasets supporting this article have been uploaded as part of the electronic supplementary material.
