# Supplementary material for: Ecological and socio-economic functions across tropical land use systems after rainforest conversion
Source: Philos Trans R Soc Lond B Biol Sci. 2016 May 19;371(1694):20150275. doi: 10.1098/rstb.2015.0275 (PMC4843696; doi:10.1098/rstb.2015.0275)
Supplement: Electronic Supplemental Material (ESM) [file rstb20150275supp1.pdf]

Electronic Supplementary Material (ESM)

Drescher et al., 2015, Ecological and socioeconomic functions across tropical land-use systems after rainforest conversion, *Phil. Trans. R. Soc. B* **371,** *111-222,* doi: 10.1098/2015.0275

**ESM Table S1: Geographic coordinates and land use types of core plots, meteo stations, climate tower and the biodiversity enrichment experiment.** For locations of household surveys see Faust et al. (1). BD = Bukit Duabelas landscape, HR Harapan landscape.

| Plot type | Landscape | Code | Land-use | Latitude | Longitude |
|---|---|---|---|---|---|
| Core plot | BD | BF1 | Forest | S 01°59'42.5" | E 102°45'08.1" |
| Core plot | BD | BF2 | Forest | S 01°58'55.1" | E 102°45'02.7" |
| Core plot | BD | BF3 | Forest | S 01°56'33.9" | E 102°34'52.7" |
| Core plot | BD | BF4 | Forest | S 01°56'31.0" | E 102°34'50.3" |
| Core plot | BD | BJ2 | Jungle rubber | S 02°01'49.7" | E 102°46'16.7" |
| Core plot | BD | BJ3 | Jungle rubber | S 02°03'46.7" | E 102°48'03.5" |
| Core plot | BD | BJ4 | Jungle rubber | S 02°00'57.3" | E 102°45'12.3" |
| Core plot | BD | BJ5 | Jungle rubber | S 02°08'35.6" | E 102°51'04.7" |
| Core plot | BD | BJ6 | Jungle rubber | S 02°01'49.3" | E 102°46'15.0" |
| Core plot | BD | BR1 | Rubber | S 02°05'30.7" | E 102°48'30.7" |
| Core plot | BD | BR2 | Rubber | S 02°05'06.8" | E 102°47'20.7" |
| Core plot | BD | BR3 | Rubber | S 02°05'43.0" | E 102°46'59.6" |
| Core plot | BD | BR4 | Rubber | S 02°04'36.1" | E 102°46'22.3" |
| Core plot | BD | BO2 | Oil palm | S 02°04'32.0" | E 102°47'30.7" |
| Core plot | BD | BO3 | Oil palm | S 02°04'15.2" | E 102°47'30.6" |
| Core plot | BD | BO4 | Oil palm | S 02°03'01.5" | E 102°45'12.1" |
| Core plot | BD | BO5 | Oil palm | S 02°06'48.9" | E 102°47'44.5" |
| Core plot | HR | HF1 | Forest | S 02°09'09.9" | E 103°21'43.2" |
| Core plot | HR | HF2 | Forest | S 02°09'29.4" | E 103°20'01.5" |
| Core plot | HR | HF3 | Forest | S 02°10'30.1" | E 103°19'57.8" |
| Core plot | HR | HF4 | Forest | S 02°11'15.2" | E 103°20'33.4" |
| Core plot | HR | HJ1 | Jungle rubber | S 01°55'40.0" | E 103°15'33.8" |
| Core plot | HR | HJ2 | Jungle rubber | S 01°49'31.9" | E 103°17'39.2" |
| Core plot | HR | HJ3 | Jungle rubber | S 01°50'56.9" | E 103°17'59.9" |
| Core plot | HR | HJ4 | Jungle rubber | S 01°47'07.3" | E 103°16'36.9" |
| Core plot | HR | HR1 | Rubber | S 01°54'39.5" | E 103°16'00.1" |
| Core plot | HR | HR2 | Rubber | S 01°52'44.5" | E 103°16'28.4" |
| Core plot | HR | HR3 | Rubber | S 01°51'34.8" | E 103°18'02.1" |
| Core plot | HR | HR4 | Rubber | S 01°48'18.2" | E 103°15'52.0" |
| Core plot | HR | HO1 | Oil palm | S 01°54'35.6" | E 103°15'58.3" |
| Core plot | HR | HO2 | Oil palm | S 01°53'00.7" | E 103°16'03.6" |
| Core plot | HR | HO3 | Oil palm | S 01°51'28.4" | E 103°18'27.4" |
| Core plot | HR | HO4 | Oil palm | S 01°47'12.7" | E 103°16'14.0" |
| Enrich. Exp.. | HR | PT Humusindo | Oil palm | S 01°56'30.7" | E 103°15'6.67" |
| Meteo-station | HR | Bungku | Village | S 01°54'06.5" | E 103°15'08.0" |
| Meteo-station | HR | PT REKI | Forest | S 02°07'34.2" | E 103°22'08.5" |
| Meteo-station | BD | L. Kepayang | Village | S 02°05'17.6" | E 102°46'03.1" |
| Meteo-station | BD | P. Kabau | Village | S 01°57'58.7" | E 102°36'05.1" |
| Climate Tower | HR | PTPN VI | 12yr oil palm | S 01°41'35.0" | E 103°23'29.0" |
| Climate Tower | HR | Pompa Air | 2yr oil palm | S 01°50'7.6" | E103°17'44.2" |

Drescher et al., 2015, Ecological and socioeconomic functions across tropical land-use systems after rainforest conversion, *Phil. Trans. R. Soc. B* **371,** *111-222,* doi: 10.1098/2015.0275

**ESM Figure S1: Core plot design.**

Five nested 5 x 5 m subplots are located in fixed positions within the 50 x 50 m core. A meteorological station is installed in the centre of each plot that measures hourly air temperature, relative air humidity at 2 m above ground and soil temperature and moisture at 0.3 m depth.

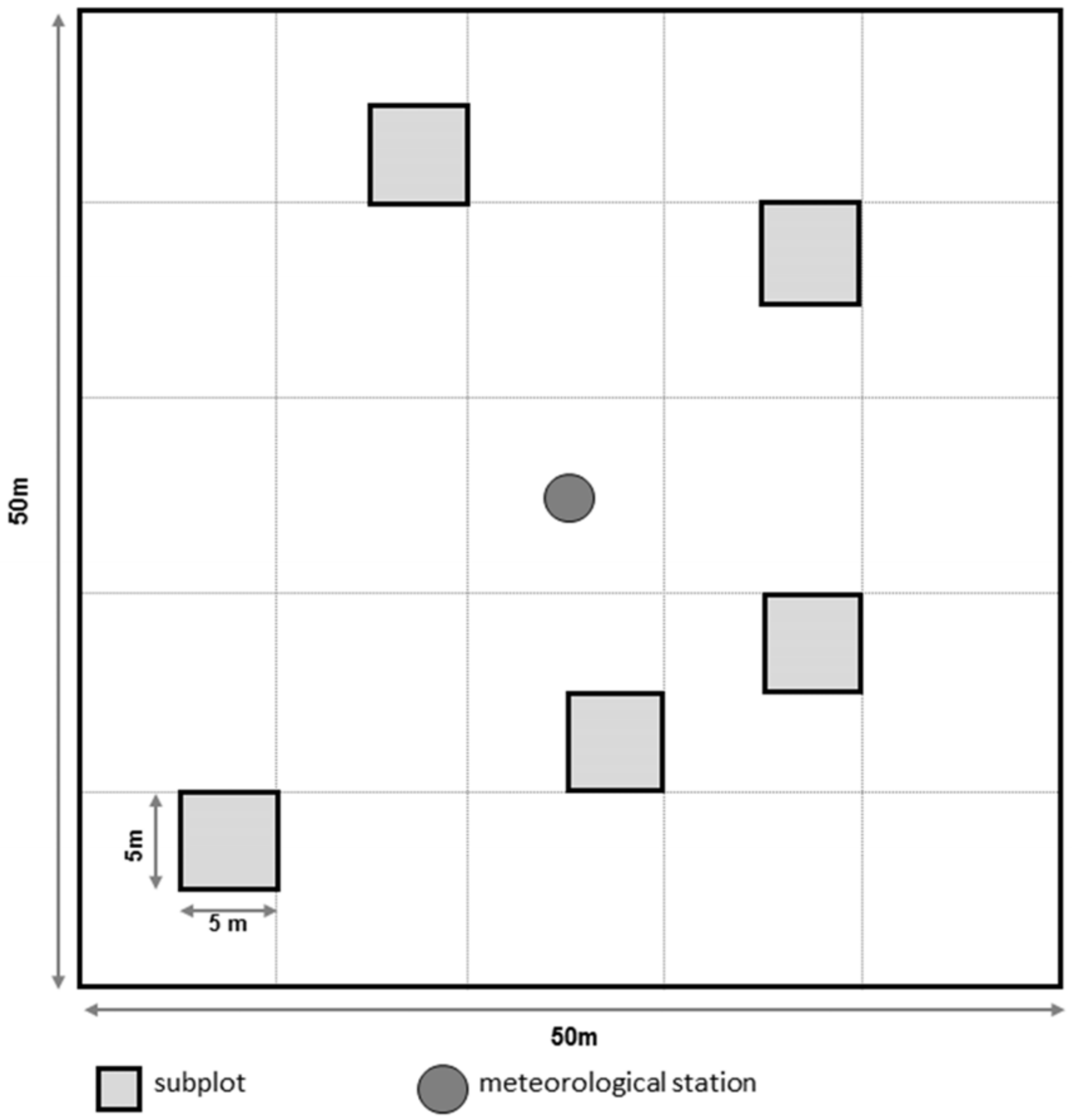

Drescher et al., 2015, Ecological and socioeconomic functions across tropical land-use systems after rainforest conversion, *Phil. Trans. R. Soc. B* **371,** *111-222,* doi: 10.1098/2015.0275

**ESM Figure S2: Sampling framework of the socioeconomic survey.**

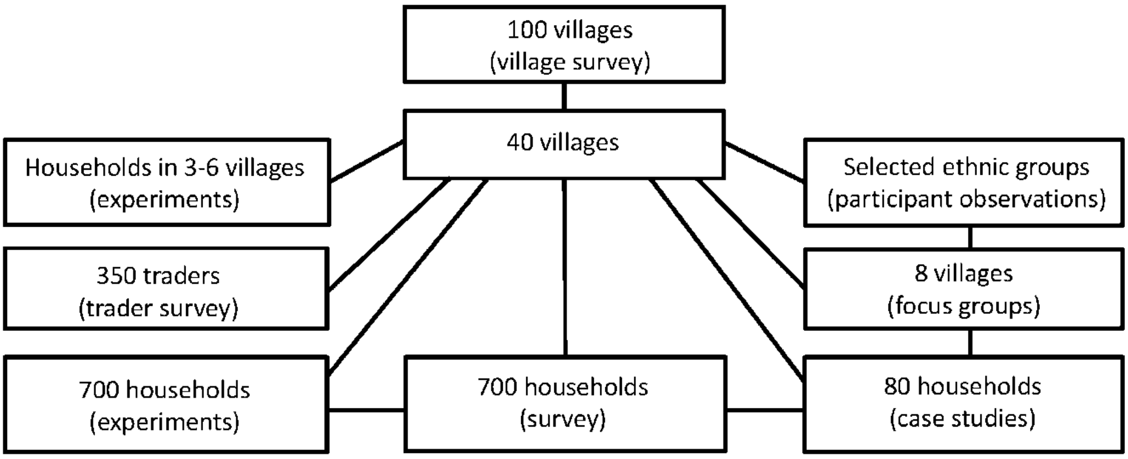

## ESM Text S1: Additional methods and results

***Evaluation of land-use changes 1990-2013*** – Land-use/land-cover (LULC) maps produced for the years of 1990, 2000, 2011, and 2013 were prepared by The Forest Resources Inventory and Remote Sensing Laboratory of the Forestry Faculty at Bogor Agricultural University (IPB). Time series of Landsat imagery with spatial resolution at 30 x 30 m of TM 1989-1990, TM/ETM+ 1999-2001, TM/ETM+ 2009-2011, and OLI 2013 were used as data sources (TM: Thematic Mapper, ETM+: Enhanced Thematic Mapper Plus, OLI: Operational Land Imager). Visual interpretation by on-screen digitation was conducted to produce different LULC classes based on the Landsat image mosaics after a pre-processing image procedure including image to image rectification and image enhancement for each acquisition. Visual interpretation was based on the LULC classification guideline produced by the Indonesian Ministry of Forestry (2) with 23 classes. During visual interpretation, local knowledge on LULC classes and RapidEye images with higher spatial resolution (5 × 5 m) at particular regions within the study area were used additionally. The 2013 LULC maps were validated using 298 reference points taken from different classes in 2014 resulting in an overall accuracy assessment of 78.2%.

***Tree heterogeneity*** – To test for environmental heterogeneity in the core plots, we used the abundance of large trees (DBH ≥ 10 cm) of all species as an indicator of habitat quality to capture strong environmental habitat factors common to all species (3). For each plot, we tested for significant deviation from homogeneity based on 199 realizations of the complete spatial randomness null model (i.e. a homogeneous spatial Poisson process). Observed field data and null models were illustrated and contrasted using the g-function, the L-function, and a goodness-of-fit test (GoF; distance interval 20 m to 25 m, cutoff point 0.05) using Programita, version January 2014 (4). While trees are homogeneously distributed at scales beyond the planting distance in rubber and oil palm plantations, most forest and jungle rubber plots were homogeneous, with the exception of one forest plot in the Harapan landscape (HF2: $p = 2.0\%$) and one jungle rubber plot each in the Bukit Duabelas (BJ5: $p = 0.5\%$) and Harapan (HJ2: $p = 1.5\%$) landscapes.

## ESM Text S2: Biodiversity Enrichment Experiment and Meteorological Monitoring

***Biodiversity Enrichment Experiment*** – The biodiversity enrichment experiment was established in December 2013 within the oil palm plantation of PT Humusindo in the Harapan landscape (Main Document, Fig. 2). To investigate the effects of increased tree biodiversity on ecological and economic performance of palm oil plantations, six native tree species were planted in gaps between oil palms: *Parkia speciosa, Durio zibethinus, Archidendron pauciflorum, Peronema canescens, Shorea leprosula, Dyera polyphylla*. These species are economically relevant for timber, fruit or latex. Plots of 5 × 5 m, 10 × 10 m, 20 × 20 m, and 40 × 40 m were established adopting a random partitions design (5), resulting in a total of 52 experimental plots with different tree species compositions. The experiment aims to reveal the spatial and temporal effects of biodiversity and ecosystem functions on gaps and the surrounding oil palm and helps to understand the socio-economic benefits and constraints of enrichment planting. The longer term goal of this experiment is to evaluate whether such enrichment planting might be developed towards a promising option to enhance the ecological value of oil palm plantations without significantly compromising their economic performance.

***Meteorological Monitoring Network*** – In addition to the 32 meteo-stations located within the 32 core plots, two meteorological stations with extended instrumentation were installed in each of the two landscapes (in proximity to forest and to agricultural systems respectively, Main Document, Fig. 2). These four meteo-stations measure rainfall, wind direction and speed, radiation (global radiation, net radiation, photosynthetically active radiation), as well as air temperature, air humidity, soil temperature, soil moisture and soil heat fluxes. Furthermore, we installed a climate tower, initially in a 2 year old oil palm plantation (Pompa Air, Fig. 2) and subsequently moved to a mature plantation (PTPN VI, Fig. 2). The climate tower allows continuous quantification of net fluxes of water, energy and greenhouse gases ($CO_2$ and $CH_4$) (6) exchanged between oil palm plantation and atmosphere, integrating both soil and canopy sources and sinks.

Drescher et al., 2015, Ecological and socioeconomic functions across tropical land-use systems after rainforest conversion, *Phil. Trans. R. Soc. B* **371,** *111-222,* doi: 10.1098/2015.0275

## Supplementary Table S2. Data for environmental and biological results in Figure 5.

Abbreviations: F – Rainforest, J - Jungle Rubber, R - Rubber plantation, O - Oil Palm plantation.

| Core Plot | System | Mean Temp. [°C] | Mean Humidity [%] | Canopy Openness [%] | Litterfall [Mg/ha/yr] | Litter Carbon [g/m²] | Tree Biomass [Mg/ha] | Plant species [N] | Ant species [N] | Oribatid mite species [N] |
|---|---|---|---|---|---|---|---|---|---|---|
| BF1 | F | 24.50 | 97.249 | 2.36 | 6.86 | 78.39 | 131.41 | 278 | 68 | NA |
| BF2 | F | 24.48 | 98.615 | 3.35 | 5.83 | 69.64 | 163.55 | 279 | 62 | NA |
| BF3 | F | 24.42 | 96.013 | 2.04 | 9.86 | 71.95 | 176.69 | 193 | 75 | NA |
| BF4 | F | 24.85 | 94.571 | 2.22 | 7.16 | 131.50 | 224.36 | 203 | 49 | NA |
| BJ2 | J | 25.00 | 94.940 | NA | NA | 39.63 | NA | NA | NA | NA |
| BJ3 | J | 25.42 | 91.383 | 6.39 | 5.20 | 62.31 | 78.18 | 169 | 56 | NA |
| BJ4 | J | 24.92 | 94.560 | 5 | 7.73 | 50.60 | 76.57 | 205 | 50 | NA |
| BJ5 | J | 25.25 | 93.318 | 5.85 | 5.36 | 56.35 | 85.02 | 168 | 40 | NA |
| BJ6 | J | NA | NA | 4.69 | 6.77 | NA | 81.80 | 160 | 42 | NA |
| BR1 | R | 25.45 | 91.287 | 14.49 | 3.08 | 31.51 | 47.03 | 58 | 25 | NA |
| BR2 | R | 25.53 | 89.461 | 12.68 | 2.90 | 34.53 | 25.98 | 52 | 20 | NA |
| BR3 | R | 25.78 | 86.896 | 10.9 | 3.48 | 48.01 | 24.43 | 44 | 27 | NA |
| BR4 | R | 25.64 | 89.755 | 19.43 | 3.67 | 68.32 | 30.07 | 58 | 21 | NA |
| BO2 | O | 25.95 | 88.755 | 23.41 | 5.10 | 6.71 | 22.01 | 65 | 26 | NA |
| BO3 | O | 25.12 | 92.921 | 13.29 | 8.81 | 9.42 | 23.66 | 73 | 24 | NA |
| BO4 | O | 25.45 | 91.421 | 12.97 | 5.98 | 10.00 | 20.78 | 75 | 32 | NA |
| BO5 | O | 25.67 | 90.855 | 20.61 | 9.06 | 21.07 | 20.60 | 79 | 18 | NA |
| HF1 | F | 24.88 | 96.394 | 2.37 | 6.56 | 32.52 | 204.12 | 315 | 50 | 36 |
| HF2 | F | 24.91 | 95.108 | 2.56 | 6.62 | 44.72 | 174.43 | 294 | 47 | 16 |
| HF3 | F | 24.67 | 98.098 | 2.22 | 7.40 | 53.42 | 206.03 | 333 | 35 | 18 |
| HF4 | F | 24.92 | 95.466 | 3.19 | 5.76 | 72.34 | 234.57 | 301 | 43 | 41 |
| HJ1 | J | 25.37 | 91.907 | 11.39 | 6.69 | 27.09 | 68.05 | 128 | 19 | 26 |
| HJ2 | J | 25.06 | 93.752 | 6.85 | 6.96 | 35.56 | 60.35 | 130 | 33 | 29 |
| HJ3 | J | 25.13 | 91.504 | 6.76 | 5.16 | 45.27 | 64.95 | 131 | 40 | 20 |
| HJ4 | J | 25.13 | 93.609 | 7.48 | 5.74 | 63.97 | 63.03 | 179 | 44 | 35 |
| HR1 | R | 25.37 | 92.245 | 8.18 | 2.89 | 7.45 | 44.62 | 73 | 20 | 13 |
| HR2 | R | 25.62 | 90.014 | 14.76 | 3.39 | 14.64 | 30.72 | 72 | 24 | 30 |
| HR3 | R | 25.40 | 91.158 | 15.72 | 2.81 | 23.40 | 41.14 | 44 | 24 | 12 |
| HR4 | R | 25.59 | 90.404 | 11.52 | 2.47 | 29.98 | 56.58 | 92 | 22 | 14 |
| HO1 | O | 25.35 | 91.120 | 15.20 | 5.79 | 3.24 | 34.26 | 96 | 38 | 16 |
| HO2 | O | 25.49 | 91.278 | 11.80 | 5.32 | 3.79 | 33.15 | 45 | 21 | 4 |
| HO3 | O | 25.18 | 91.235 | 11.95 | 5.73 | 5.04 | 26.52 | 51 | 23 | 9 |
| HO4 | O | 25.39 | 90.944 | 15.91 | 4.99 | 5.27 | 22.93 | 85 | 19 | 7 |

Drescher et al., 2015, Ecological and socioeconomic functions across tropical land-use systems after rainforest conversion, *Phil. Trans. R. Soc. B* **371***, 111-222,* doi: 10.1098/2015.0275

**Supplementary Table S3: Data for socio-economic results in Figure 5.**

Abbreviations: J – Jungle Rubber, R – Rubber Monoculture, O – Oil Palm Monoculture.

Units: Labour ['00 hours/ha/year], gross/year [million IDR/ha/year], gross/labour ['000 IDR/hours].

| system | labour | gross/ year | gross/ labour | system | labour | gross/ year | gross/ labour | system | labour | gross/ year | gross/ labour |
|---|---|---|---|---|---|---|---|---|---|---|---|
| J | 1.98 | 3.36 | 0.00 | O | 0.75 | 1.14 | NA | O | 0.13 | -1.19 | -0.98 |
| J | 5.44 | 4.16 | 0.00 | O | 0.96 | -1.83 | NA | O | 3.66 | 25.01 | -0.96 |
| J | 9.04 | 7.65 | 0.35 | O | 0.64 | 9.33 | -18.00 | O | 2.7 | 3.33 | -0.91 |
| J | 10 | 22.01 | 0.35 | O | 1.02 | 15.93 | -16.56 | O | 11.4 | 22.08 | -0.90 |
| J | 3.79 | 1.33 | 0.40 | O | 0.16 | -1.55 | -13.54 | O | 2.61 | 15.28 | -0.83 |
| J | 11.43 | 9.77 | 0.43 | O | 1.44 | 5.86 | -11.75 | O | 1.44 | -3.40 | -0.62 |
| J | 3.36 | 11.60 | 0.48 | O | 2.11 | 5.97 | -10.67 | O | 3.82 | -7.24 | -0.49 |
| J | 3.86 | 5.92 | 0.48 | O | 1.49 | 21.22 | -9.65 | O | 1.08 | 37.07 | -0.45 |
| J | 4.9 | 15.76 | 0.50 | O | 1.69 | 14.03 | -9.43 | O | 4.68 | 12.82 | -0.45 |
| J | 3.65 | 3.13 | 0.52 | O | 1.84 | 4.87 | -9.35 | O | 1.45 | -7.31 | -0.41 |
| J | 7.84 | 3.15 | 0.60 | O | 0.14 | -0.42 | -9.33 | O | 2.43 | 15.13 | -0.34 |
| J | 13.72 | 21.13 | 0.61 | O | 0.79 | 25.88 | -9.09 | O | 1.06 | 0.07 | -0.31 |
| J | 3.2 | 6.15 | 0.62 | O | 0.8 | 10.05 | -9.09 | O | 0.88 | 3.26 | -0.30 |
| J | 0 | 0.00 | 0.76 | O | 1.62 | 25.66 | -8.96 | O | 1.41 | -7.76 | -0.24 |
| J | 7.84 | 3.75 | 0.83 | O | 0.28 | -0.78 | -8.88 | O | 1.35 | -0.56 | -0.22 |
| J | 21.6 | 27.76 | 0.85 | O | 0.12 | -1.22 | -8.85 | O | 1.08 | -1.81 | -0.14 |
| J | 7.84 | 3.75 | 0.85 | O | 0.21 | -2.07 | -8.65 | O | 0.41 | -1.55 | -0.09 |
| J | 4.03 | 7.58 | 0.86 | O | 0.28 | -1.12 | -8.49 | O | 1.12 | 13.45 | -0.04 |
| J | 5.48 | 24.18 | 1.04 | O | 0.36 | -1.14 | -8.34 | O | 1.04 | 5.39 | -0.02 |
| J | 4.08 | 2.04 | 1.25 | O | 3.36 | -2.09 | -8.13 | O | 0.28 | -1.47 | 0.00 |
| J | 0 | 0.00 | 1.26 | O | 0.16 | -2.30 | -8.02 | O | 0.52 | 3.37 | 0.00 |
| J | 1.68 | 2.11 | 1.29 | O | 2.08 | 10.63 | -7.82 | O | 0.6 | 15.70 | 0.00 |
| J | 7.83 | 10.87 | 1.39 | O | 0.4 | 0.06 | -7.16 | O | 0.98 | 14.03 | 0.00 |
| J | 2.72 | 2.26 | 1.53 | O | 1.68 | 7.57 | -6.70 | O | 1.05 | -3.32 | 0.00 |
| J | 0.88 | 9.18 | 1.54 | O | 2.39 | 12.11 | -5.94 | O | 1.16 | 17.49 | 0.00 |
| J | 10.01 | 3.55 | 1.69 | O | 0.84 | 4.90 | -5.65 | O | 1.18 | -3.77 | 0.00 |
| J | 6.21 | 2.67 | 1.78 | O | 0.99 | 8.16 | -5.60 | O | 1.26 | 9.68 | 0.00 |
| J | 11.8 | 12.29 | 1.88 | O | 2.37 | 31.50 | -5.60 | O | 2.24 | -5.72 | 0.00 |
| J | 7.76 | 13.85 | 1.92 | O | 2.47 | 1.56 | -5.46 | O | 2.44 | 31.68 | 0.00 |
| J | 6.33 | 3.91 | 2.07 | O | 1.73 | 10.78 | -5.36 | O | 2.56 | 12.00 | 0.00 |
| J | 9.82 | 6.03 | 2.20 | O | 3.4 | -4.27 | -5.07 | O | 3.06 | 9.75 | 0.00 |
| J | 5.78 | 16.87 | 2.91 | O | 2.45 | 9.09 | -5.01 | O | 4.68 | 18.21 | 0.00 |
| J | 7.84 | 4.09 | 3.21 | O | 3.28 | 9.18 | -4.75 | O | 1.41 | 10.36 | 0.00 |
| J | 4.74 | 9.82 | 3.44 | O | 2.02 | 14.72 | -4.61 | O | 2.12 | 17.36 | 0.07 |
| J | 9.31 | 5.55 | 4.40 | O | 0.08 | -0.96 | -4.59 | O | 2.67 | 15.38 | 0.14 |
| J | 10.24 | 12.89 | 10.37 | O | 3.66 | 4.08 | -4.59 | O | 1.04 | 5.39 | 0.15 |
| | | | | O | 0.1 | -0.39 | -4.51 | O | 1.07 | 21.42 | 0.20 |
| | | | | O | 0.96 | 5.07 | -4.28 | O | 1.69 | 14.03 | 0.25 |
| | | | | O | 0 | 0.00 | -4.18 | O | 2.4 | 11.69 | 0.38 |
| | | | | O | 2.48 | 6.00 | -4.14 | O | 1.25 | -2.21 | 0.40 |
| | | | | O | 2.01 | 16.18 | -4.11 | O | 0.09 | -0.80 | 0.42 |
| | | | | O | 0.18 | -3.15 | -3.90 | O | 0.09 | -0.87 | 0.42 |
| | | | | O | 3.31 | 11.07 | -3.87 | O | 0.08 | -2.18 | 0.42 |
| | | | | O | 3.87 | 3.08 | -3.79 | O | 2.4 | 19.82 | 0.59 |

Electronic Supplementary Material (ESM)

Drescher et al., 2015, Ecological and socioeconomic functions across tropical land-use systems after rainforest conversion, *Phil. Trans. R. Soc. B* **371,** *111-222,* doi: 10.1098/2015.0275

| system | labour | gross/ year | gross/ labour | system | labour | gross/ year | gross/ labour | system | labour | gross/ year | gross/ labour |
|---|---|---|---|---|---|---|---|---|---|---|---|
| O | 2.44 | 2.56 | 0.93 | O | 2.4 | 19.82 | 0.59 | O | 1.76 | 7.52 | 2.73 |
| O | 3.36 | 14.72 | 0.95 | O | 2.56 | 6.39 | 0.60 | O | 0.89 | 23.12 | 2.76 |
| O | 1.25 | 22.89 | 1.03 | O | 0.52 | 2.72 | 0.63 | O | 0.67 | 7.68 | 2.79 |
| O | 2.37 | 33.48 | 1.05 | O | 0.12 | -0.73 | 0.75 | O | 2.6 | 5.74 | 2.80 |
| O | 2.88 | 4.74 | 1.11 | O | 1.25 | 22.89 | 0.79 | O | 1.15 | 7.94 | 2.80 |
| O | 0.74 | -1.34 | 1.15 | O | 1.33 | 1.97 | 0.79 | O | 1.14 | 3.64 | 2.82 |
| O | 1.32 | 17.46 | 1.17 | O | 1.42 | 1.17 | 0.82 | O | 1.76 | 8.95 | 2.82 |
| O | 1.19 | 7.58 | 1.23 | O | 2.44 | 2.56 | 0.93 | O | 1.14 | 19.93 | 2.86 |
| O | 1.19 | 7.58 | 1.23 | O | 3.36 | 14.72 | 0.95 | O | 2.64 | 3.43 | 2.86 |
| O | 0.13 | -1.19 | -0.98 | O | 1.25 | 22.89 | 1.03 | O | 0.57 | -2.07 | 2.90 |
| O | 3.66 | 25.01 | -0.96 | O | 2.37 | 33.48 | 1.05 | O | 0.6 | 12.73 | 2.90 |
| O | 2.7 | 3.33 | -0.91 | O | 2.88 | 4.74 | 1.11 | O | 0.9 | 5.16 | 3.00 |
| O | 11.4 | 22.08 | -0.90 | O | 0.74 | -1.34 | 1.15 | O | 1.66 | -5.91 | 3.03 |
| O | 2.61 | 15.28 | -0.83 | O | 1.32 | 17.46 | 1.17 | O | 0.08 | -0.50 | 3.04 |
| O | 1.44 | -3.40 | -0.62 | O | 1.19 | 7.58 | 1.23 | O | 2.84 | -2.94 | 3.04 |
| O | 3.82 | -7.24 | -0.49 | O | 1.19 | 7.58 | 1.23 | O | 0 | -0.52 | 3.11 |
| O | 1.08 | 37.07 | -0.45 | O | 0.05 | -0.05 | 1.30 | O | 6.38 | 25.31 | 3.13 |
| O | 4.68 | 12.82 | -0.45 | O | 0.48 | -3.98 | 1.30 | O | 1.25 | 18.02 | 3.17 |
| O | 1.45 | -7.31 | -0.41 | O | 0.24 | -0.95 | 1.33 | O | 2.19 | 7.29 | 3.17 |
| O | 2.43 | 15.13 | -0.34 | O | 3.08 | 14.96 | 1.39 | O | 2.26 | -10.46 | 3.18 |
| O | 1.06 | 0.07 | -0.31 | O | 8.15 | -2.48 | 1.40 | O | 1.8 | 9.64 | 3.19 |
| O | 0.88 | 3.26 | -0.30 | O | 0.12 | -0.46 | 1.45 | O | 5.32 | 9.10 | 3.21 |
| O | 1.41 | -7.76 | -0.24 | O | 2.9 | 3.78 | 1.47 | O | 0.88 | 5.24 | 3.31 |
| O | 1.35 | -0.56 | -0.22 | O | 1.21 | 7.80 | 1.51 | O | 1.68 | -2.06 | 3.33 |
| O | 1.08 | -1.81 | -0.14 | O | 0.74 | -2.77 | 1.58 | O | 0.1 | -1.03 | 3.34 |
| O | 0.41 | -1.55 | -0.09 | O | 0.64 | 1.61 | 1.61 | O | 1.62 | 4.67 | 3.37 |
| O | 1.12 | 13.45 | -0.04 | O | 0.54 | -1.95 | 1.64 | O | 1.8 | 4.08 | 3.57 |
| O | 1.04 | 5.39 | -0.02 | O | 8.33 | -2.61 | 1.71 | O | 0.1 | -0.45 | 3.59 |
| O | 0.28 | -1.47 | 0.00 | O | 0.48 | -1.91 | 1.83 | O | 3.42 | -0.48 | 3.65 |
| O | 0.52 | 3.37 | 0.00 | O | 0.2 | -0.48 | 1.85 | O | 2.37 | 29.66 | 3.69 |
| O | 0.6 | 15.70 | 0.00 | O | 2.9 | -5.80 | 1.94 | O | 1.8 | 18.63 | 3.71 |
| O | 0.98 | 14.03 | 0.00 | O | 0.79 | 2.07 | 1.96 | O | 0.22 | -0.19 | 3.77 |
| O | 1.05 | -3.32 | 0.00 | O | 1.6 | 14.01 | 1.96 | O | 4.68 | 14.95 | 3.78 |
| O | 1.16 | 17.49 | 0.00 | O | 0.18 | -1.14 | 2.01 | O | 2.22 | 31.21 | 3.86 |
| O | 1.18 | -3.77 | 0.00 | O | 1.49 | 3.99 | 2.09 | O | 1.78 | 2.89 | 3.88 |
| O | 1.26 | 9.68 | 0.00 | O | 0.67 | 0.13 | 2.11 | O | 1.29 | 5.53 | 3.96 |
| O | 2.24 | -5.72 | 0.00 | O | 0.16 | 0.25 | 2.15 | O | 1.5 | 1.13 | 3.97 |
| O | 2.44 | 31.68 | 0.00 | O | 2.37 | 7.64 | 2.17 | O | 0.48 | -2.05 | 4.01 |
| O | 2.56 | 12.00 | 0.00 | O | 2.16 | 0.00 | 2.20 | O | 2.91 | 11.60 | 4.05 |
| O | 3.06 | 9.75 | 0.00 | O | 2.22 | 21.75 | 2.25 | O | 0.7 | 3.33 | 4.07 |
| O | 4.68 | 18.21 | 0.00 | O | 2.4 | 21.10 | 2.26 | O | 2.69 | 26.84 | 4.14 |
| O | 1.41 | 10.36 | 0.00 | O | 0.96 | 13.58 | 2.41 | O | 2.69 | 26.84 | 4.14 |
| O | 2.12 | 17.36 | 0.07 | O | 1.07 | 18.32 | 2.46 | O | 5.88 | 9.31 | 4.16 |
| O | 2.67 | 15.38 | 0.14 | O | 0 | 0.00 | 2.49 | O | 4.68 | 13.23 | 4.17 |
| O | 1.04 | 5.39 | 0.15 | O | 2.27 | 13.34 | 2.51 | O | 2.15 | 29.12 | 4.20 |
| O | 1.07 | 21.42 | 0.20 | O | 1.99 | 12.00 | 2.53 | O | 3.2 | 32.13 | 4.24 |
| O | 1.69 | 14.03 | 0.25 | O | 1.22 | -0.04 | 2.61 | O | 0.26 | -0.24 | 4.25 |
| O | 2.4 | 11.69 | 0.38 | O | 0 | 0.00 | 2.61 | O | 0.4 | -4.82 | 4.31 |
| O | 1.25 | -2.21 | 0.40 | O | 0.84 | -2.73 | 2.63 | O | 0 | 0.00 | 4.33 |

| system | labour | gross/ year | gross/ labour | system | labour | gross/ year | gross/ labour | system | labour | gross/ year | gross/ labour |
|---|---|---|---|---|---|---|---|---|---|---|---|
| O | 0.09 | -0.80 | 0.42 | O | 1.8 | 12.14 | 2.65 | O | 2.2 | 9.21 | 4.36 |
| O | 0.09 | -0.87 | 0.42 | O | 0 | -0.18 | 2.65 | O | 0.69 | 1.30 | 4.37 |
| O | 0.08 | -2.18 | 0.42 | O | 4.68 | 12.41 | 2.68 | O | 2.1 | 0.00 | 4.41 |
| O | 3.2 | 8.98 | 4.48 | O | 3.13 | 0.78 | 5.14 | O | 0.62 | -2.11 | 8.28 |
| O | 0 | 0.00 | 4.64 | O | 0.68 | -3.17 | 5.15 | O | 0.64 | 1.36 | 8.56 |
| O | 2.45 | 16.58 | 4.67 | O | 1.16 | -1.15 | 5.23 | O | 1.7 | 27.02 | 8.70 |
| O | 1.62 | -0.40 | 4.69 | O | 1.58 | 11.41 | 5.33 | O | 1.52 | 21.18 | 8.75 |
| O | 3.14 | 13.91 | 4.72 | O | 2.96 | 10.65 | 5.40 | O | 2.7 | 3.33 | 8.77 |
| O | 0.73 | -0.78 | 4.75 | O | 1 | 3.90 | 5.67 | O | 0.67 | 7.37 | 8.92 |
| O | 0.36 | -2.65 | 4.78 | O | 0.36 | -0.67 | 5.68 | O | 0.56 | 1.23 | 9.30 |
| O | 1.5 | 0.00 | 4.84 | O | 1.67 | -0.75 | 5.74 | O | 2.08 | 5.97 | 9.75 |
| O | 0.65 | 9.41 | 4.85 | O | 2.36 | 15.38 | 5.76 | O | 0.92 | 4.06 | 9.94 |
| O | 1.84 | 3.62 | 4.85 | O | 2.2 | 9.00 | 5.83 | O | 0.93 | 11.22 | 9.94 |
| O | 2.22 | 27.04 | 5.02 | O | 0.88 | 7.18 | 5.84 | O | 1.86 | 11.34 | 10.01 |
| O | 2.08 | 14.60 | 5.04 | O | 3.44 | 14.47 | 5.86 | O | 1.87 | 2.50 | 10.07 |
| O | 0.61 | -1.32 | 5.06 | O | 2.25 | 6.22 | 6.00 | O | 0 | 0.00 | 10.24 |
| O | 3.18 | 9.71 | 5.06 | O | 2.37 | 34.90 | 6.06 | O | 0.86 | 4.96 | 10.30 |
| O | 2.16 | 23.03 | 5.09 | O | 2.34 | 9.41 | 6.19 | O | 0 | 0.00 | 10.50 |
| O | 2.5 | 12.67 | 5.13 | O | 0 | 0.00 | 6.19 | O | 1.88 | 17.58 | 10.61 |
| O | 3.12 | 1.32 | 5.14 | O | 0.9 | -4.11 | 6.20 | O | 1.7 | 1.63 | 10.78 |
| O | 3.13 | 0.78 | 5.14 | O | 2.34 | 4.96 | 6.30 | O | 2.82 | -6.28 | 10.81 |
| O | 0.68 | -3.17 | 5.15 | O | 0.34 | -3.34 | 6.32 | O | 2.13 | 0.86 | 10.95 |
| O | 1.16 | -1.15 | 5.23 | O | 0.35 | -1.69 | 6.32 | O | 0.12 | 0.00 | 11.09 |
| O | 1.58 | 11.41 | 5.33 | O | 2.26 | 7.58 | 6.40 | O | 2.72 | 0.42 | 11.26 |
| O | 2.96 | 10.65 | 5.40 | O | 0.28 | -0.99 | 6.43 | O | 1.52 | -0.75 | 11.34 |
| O | 1 | 3.90 | 5.67 | O | 1.52 | -3.31 | 6.44 | O | 1.8 | 0.68 | 11.90 |
| O | 0.36 | -0.67 | 5.68 | O | 1.6 | 44.32 | 6.49 | O | 3.71 | -0.35 | 11.97 |
| O | 1.67 | -0.75 | 5.74 | O | 1.6 | 12.49 | 6.53 | O | 1.66 | 0.69 | 12.12 |
| O | 2.36 | 15.38 | 5.76 | O | 2.86 | 5.23 | 6.55 | O | 0.16 | -0.40 | 12.40 |
| O | 2.2 | 9.00 | 5.83 | O | 4.32 | 13.15 | 6.57 | O | 1.98 | -11.24 | 12.46 |
| O | 0.88 | 7.18 | 5.84 | O | 1.52 | 11.88 | 6.59 | O | 1.6 | 4.83 | 12.93 |
| O | 3.2 | 32.13 | 4.24 | O | 1.82 | 8.70 | 6.71 | O | 1.81 | -1.88 | 13.13 |
| O | 0.26 | -0.24 | 4.25 | O | 1.86 | 7.05 | 6.74 | O | 1.96 | 9.87 | 13.24 |
| O | 0.4 | -4.82 | 4.31 | O | 2.22 | 31.51 | 6.82 | O | 0.89 | 26.61 | 13.29 |
| O | 0 | 0.00 | 4.33 | O | 0.3 | -0.64 | 6.85 | O | 0.12 | -0.60 | 13.47 |
| O | 2.2 | 9.21 | 4.36 | O | 5.89 | 8.26 | 7.00 | O | 2.04 | 9.94 | 13.85 |
| O | 0.69 | 1.30 | 4.37 | O | 0.13 | -1.14 | 7.18 | O | 1.76 | 11.40 | 13.91 |
| O | 2.1 | 0.00 | 4.41 | O | 1.68 | 5.26 | 7.23 | O | 1.64 | 10.88 | 13.99 |
| O | 3.2 | 8.98 | 4.48 | O | 4.68 | 17.39 | 7.25 | O | 2.99 | 12.43 | 14.00 |
| O | 0 | 0.00 | 4.64 | O | 1.24 | 3.91 | 7.28 | O | 1.94 | -0.04 | 14.07 |
| O | 2.45 | 16.58 | 4.67 | O | 1.47 | 12.70 | 7.46 | O | 1.66 | 0.69 | 14.13 |
| O | 1.62 | -0.40 | 4.69 | O | 1.14 | 8.71 | 7.57 | O | 1.48 | 11.97 | 14.17 |
| O | 3.14 | 13.91 | 4.72 | O | 2.26 | -2.17 | 7.62 | O | 2.34 | 18.23 | 14.18 |
| O | 0.73 | -0.78 | 4.75 | O | 1.6 | 2.23 | 7.75 | O | 2.16 | 17.98 | 14.26 |
| O | 0.36 | -2.65 | 4.78 | O | 2.29 | 1.38 | 7.76 | O | 2.03 | 6.18 | 14.33 |
| O | 1.5 | 0.00 | 4.84 | O | 4.76 | 18.02 | 7.77 | O | 1.14 | 1.33 | 14.36 |
| O | 0.65 | 9.41 | 4.85 | O | 0.95 | -0.22 | 7.83 | O | 1.96 | 4.42 | 14.67 |
| O | 1.84 | 3.62 | 4.85 | O | 1.26 | 11.14 | 8.01 | O | 6.59 | 47.72 | 14.95 |
| O | 2.22 | 27.04 | 5.02 | O | 1.88 | 5.48 | 8.06 | O | 1.63 | 7.85 | 15.49 |

| system | labour | gross/ year | gross/ labour | system | labour | gross/ year | gross/ labour | system | labour | gross/ year | gross/ labour |
|---|---|---|---|---|---|---|---|---|---|---|---|
| O | 2.08 | 14.60 | 5.04 | O | 2.43 | -1.09 | 8.07 | O | 6.34 | 7.46 | 15.78 |
| O | 0.61 | -1.32 | 5.06 | O | 0.98 | 13.83 | 8.16 | O | 1.88 | 5.48 | 15.80 |
| O | 3.18 | 9.71 | 5.06 | O | 0.36 | -0.96 | 8.16 | O | 1.8 | -0.62 | 17.04 |
| O | 2.16 | 23.03 | 5.09 | O | 0.42 | 10.03 | 8.22 | O | 2.19 | -3.31 | 17.33 |
| O | 2.5 | 12.67 | 5.13 | O | 0 | 0.00 | 8.27 | O | 1.75 | 23.38 | 18.20 |
| O | 3.12 | 1.32 | 5.14 | O | 0 | 0.00 | 8.27 | O | 1.75 | 19.96 | 18.20 |
| R | 0.37 | 1.02 | -7.56 | R | 24.39 | 14.24 | 0.58 | R | 6.62 | 12.36 | 1.37 |
| R | 10.95 | 9.05 | -3.92 | R | 5.28 | 22.50 | 0.60 | R | 5.49 | 6.16 | 1.37 |
| R | 4.35 | 23.22 | -3.76 | R | 6.06 | 34.00 | 0.64 | R | 7.23 | 4.96 | 1.39 |
| R | 6.27 | -4.21 | -3.39 | R | 25.32 | 8.09 | 0.64 | R | 1.88 | 0.00 | 1.39 |
| R | 8.87 | 1.86 | -2.91 | R | 4.05 | 7.01 | 0.69 | R | NA | 32.28 | 1.41 |
| R | 7.74 | 7.71 | -2.78 | R | 12.59 | 32.97 | 0.70 | R | 18.24 | 25.40 | 1.43 |
| R | 16.06 | 16.90 | -2.72 | R | 0.06 | -0.20 | 0.71 | R | 5.76 | 23.50 | 1.44 |
| R | 0.1 | -0.20 | -2.44 | R | 4.16 | 7.49 | 0.78 | R | 5.97 | 19.13 | 1.44 |
| R | 30 | 18.00 | -2.41 | R | 7.08 | 8.15 | 0.79 | R | 7.32 | 19.63 | 1.45 |
| R | 7.63 | 11.01 | -2.02 | R | 10.92 | 7.04 | 0.80 | R | 0.03 | -0.10 | 1.48 |
| R | 11.22 | 11.24 | -1.82 | R | 0.4 | 0.00 | 0.82 | R | 14.8 | 38.18 | 1.48 |
| R | 8.61 | 16.61 | -1.71 | R | 4.3 | 17.25 | 0.82 | R | 3.6 | 0.00 | 1.50 |
| R | 7.17 | 10.77 | -1.33 | R | 0.1 | -0.27 | 0.83 | R | 1.15 | 2.03 | 1.54 |
| R | 22.08 | 25.85 | -1.29 | R | 9.16 | 22.86 | 0.84 | R | 6.84 | 1.23 | 1.58 |
| R | 33.6 | 29.12 | -1.26 | R | 13.3 | 48.08 | 0.85 | R | 0.18 | -0.32 | 1.62 |
| R | 16.74 | 23.57 | -1.01 | R | 13.02 | 10.65 | 0.87 | R | 15.88 | 14.45 | 1.62 |
| R | 6.78 | 20.36 | -0.98 | R | 9.93 | 21.85 | 0.88 | R | 0.3 | -1.05 | 1.68 |
| R | 4.06 | 28.62 | -0.67 | R | 6.72 | 9.20 | 0.91 | R | 8.54 | 18.03 | 1.73 |
| R | 13.7 | 21.63 | -0.40 | R | 0.54 | -0.69 | 0.91 | R | 0.18 | -0.19 | 1.74 |
| R | 5.58 | 28.52 | -0.38 | R | 3.92 | -1.50 | 0.91 | R | 6.58 | 8.46 | 1.74 |
| R | 6.82 | 9.35 | -0.35 | R | 12.24 | 15.73 | 0.92 | R | 14.35 | 10.08 | 1.80 |
| R | 4.06 | 40.34 | -0.30 | R | 0 | 0.00 | 0.94 | R | 3.75 | 17.83 | 1.82 |
| R | 5.74 | 10.59 | -0.07 | R | 9.7 | 32.98 | 0.94 | R | 15.49 | 14.08 | 1.82 |
| R | 1.57 | -2.11 | 0.00 | R | 14.29 | 32.42 | 0.95 | R | 8.85 | 13.11 | 1.84 |
| R | 5.62 | 7.83 | 0.00 | R | 5.13 | 15.00 | 0.98 | R | 10.68 | 25.70 | 1.86 |
| R | 5.62 | 8.15 | 0.00 | R | 6.75 | 7.09 | 1.00 | R | 6.37 | 12.76 | 1.89 |
| R | 5.68 | 10.34 | 0.00 | R | 9.23 | 14.93 | 1.00 | R | 6.38 | 15.16 | 1.89 |
| R | 6.28 | 21.12 | 0.00 | R | 6.19 | 6.60 | 1.00 | R | 6.42 | 9.53 | 1.89 |
| R | 7.57 | 31.98 | 0.00 | R | 5.62 | 2.08 | 1.01 | R | 12.48 | 16.81 | 1.93 |
| R | 8.1 | -2.46 | 0.00 | R | 8.74 | 11.95 | 1.05 | R | 0.24 | -0.60 | 1.98 |
| R | 8.98 | 4.90 | 0.00 | R | 9.52 | -0.65 | 1.05 | R | 21.24 | 4.90 | 2.00 |
| R | 9.5 | 22.04 | 0.00 | R | 7.9 | 16.18 | 1.06 | R | 0 | 0.00 | 2.00 |
| R | 19.56 | 21.44 | 0.00 | R | 16.97 | 4.88 | 1.10 | R | 10.77 | 20.32 | 2.05 |
| R | 19.58 | 19.49 | 0.00 | R | 7.33 | 8.35 | 1.10 | R | 13.64 | 17.68 | 2.05 |
| R | 19.6 | 23.19 | 0.00 | R | 9.3 | 10.99 | 1.12 | R | 6.22 | 32.16 | 2.06 |
| R | 9.23 | 16.05 | 0.18 | R | 21.22 | 41.99 | 1.14 | R | 6.91 | 6.32 | 2.08 |
| R | 1.34 | -1.32 | 0.21 | R | 7.43 | 9.44 | 1.15 | R | 19.87 | 12.68 | 2.11 |
| R | 8.92 | 13.76 | 0.23 | R | 2.51 | 32.46 | 1.16 | R | 17.17 | 36.26 | 2.11 |
| R | 7.16 | 4.00 | 0.23 | R | 0.16 | -1.29 | 1.17 | R | 11.48 | 14.38 | 2.20 |
| R | 8.48 | 6.93 | 0.27 | R | 1.28 | -0.46 | 1.18 | R | 16.14 | 5.62 | 2.25 |
| R | 3.16 | 3.67 | 0.29 | R | 11.34 | 10.66 | 1.18 | R | 0 | 0.00 | 2.27 |
| R | 22.38 | 19.58 | 0.32 | R | 7.43 | 36.52 | 1.20 | R | 8.47 | 6.61 | 2.30 |
| R | 23.36 | 21.38 | 0.32 | R | 6.29 | 19.59 | 1.20 | R | 10.23 | 9.75 | 2.32 |

| system | labour | gross/ year | gross/ labour | system | labour | gross/ year | gross/ labour |
|---|---|---|---|---|---|---|---|
| R | 0.03 | -0.09 | 0.35 | R | 6.08 | 22.22 | 1.20 |
| R | 6.22 | 4.90 | 0.37 | R | 7.77 | 6.22 | 1.20 |
| R | 3.6 | 0.00 | 0.38 | R | 12.58 | 4.90 | 1.25 |
| R | 7.08 | 7.83 | 0.39 | R | 8.5 | 2.25 | 1.27 |
| R | 7.03 | 10.07 | 0.45 | R | 6.88 | 6.94 | 1.28 |
| R | 6.22 | 35.46 | 0.46 | R | 7.54 | 6.36 | 1.28 |
| R | 7 | 11.38 | 0.46 | R | 10.56 | 31.85 | 1.30 |
| R | 7.08 | 8.48 | 0.55 | R | 10.77 | 20.32 | 1.35 |
| R | 4.12 | 19.44 | 0.56 | R | 25.32 | 8.09 | 1.37 |
| R | 4.39 | 9.16 | 2.69 | R | 16.24 | 59.93 | 2.37 |
| R | 5.42 | 12.49 | 2.70 | R | 30 | 29.41 | 2.40 |
| R | 5.52 | 17.42 | 2.71 | R | 8.88 | 22.60 | 2.49 |
| R | 11.8 | 14.20 | 2.80 | R | 7.63 | 11.01 | 2.54 |
| R | 22.2 | 59.71 | 2.92 | R | 0.1 | -0.43 | 2.57 |
| R | 0 | 0.00 | 2.93 | R | 4.4 | 22.06 | 2.58 |
| R | 0.42 | -1.62 | 3.00 | R | 0 | 0.00 | 2.62 |
| R | 7.44 | 15.35 | 3.01 | R | 8.52 | 23.85 | 2.67 |
| R | 10.64 | 31.23 | 3.11 | R | 0.16 | 0.00 | 2.68 |
| R | 0 | 0.00 | 3.15 | | | | |
| R | 18.95 | 38.86 | 3.19 | | | | |
| R | 6.48 | 1.46 | 3.19 | | | | |
| R | 8.97 | 8.40 | 3.20 | | | | |
| R | 20.77 | 14.67 | 3.36 | | | | |
| R | 12.28 | 22.38 | 3.40 | | | | |
| R | 3.81 | 4.58 | 3.61 | | | | |
| R | 0.34 | -0.95 | 3.65 | | | | |
| R | 18.75 | 15.86 | 3.67 | | | | |
| R | 4.56 | 12.33 | 3.69 | | | | |
| R | 10.68 | 4.90 | 4.00 | | | | |
| R | 0.11 | -0.05 | 4.07 | | | | |
| R | 10.77 | 20.32 | 4.22 | | | | |
| R | 4.92 | 11.11 | 4.25 | | | | |
| R | 10.51 | 4.72 | 4.71 | | | | |
| R | 3.6 | 0.00 | 4.74 | | | | |
| R | 0.11 | -0.15 | 4.91 | | | | |
| R | 10.91 | 13.07 | 5.00 | | | | |
| R | 6.26 | 19.98 | 5.10 | | | | |
| R | 9.39 | 29.94 | 5.16 | | | | |
| R | 14.4 | 5.46 | 5.33 | | | | |
| R | 4.22 | 11.47 | 5.60 | | | | |
| R | 9.39 | 34.54 | 5.69 | | | | |
| R | 9.69 | 24.96 | 7.03 | | | | |
| R | 9.56 | 16.04 | 9.91 | | | | |
| R | 5.46 | 10.94 | 12.88 | | | | |

## Supplementary References

1. Faust H, Schwarze S, Beckert B, Brümmer B, Dittrich C, Euler M, et al. 2013. Assessment of socio-economic functions of tropical lowland transformation systems in Indonesia: Sampling framework and methodological approach. EFForTS discussion paper series.
2. Ministry of Forestry Indonesia 2008. Pemantauan Sumber Daya Hutan (Monitoring of Forest Resource), Jakarta. http://nfms.dephut.go.id/ipsdh/.
3. Getzin S, Wiegand T, Wiegand K, He F. 2008. Heterogeneity influences spatial patterns and demographics in forest stands. *J Ecol* **96**: 807-20. 10.1111/j.1365-2745.2008.01377.x.
4. Wiegand T, Moloney KA. Handbook of spatial point-pattern analysis in ecology. 2013.
5. Bell T, Killey AK, Hector A, Schmid B, King L, Newman JA. 2009. A Linear Model Method for Biodiversity-Ecosystem Functioning Experiments. *The American Naturalist* **174**: 836-49. 10.1086/647931.
6. Baldocchi DD. 2003. Assessing the eddy covariance technique for evaluating carbon dioxide exchange rates of ecosystems: past, present and future. *Global Change Biol* **9**: 479-92.
